# Endocardite à candida albicans compliquant une maladie de Behçet

**DOI:** 10.11604/pamj.2015.20.441.6110

**Published:** 2015-04-30

**Authors:** Madiha Mahfoudhi, Khammassi Khaled

**Affiliations:** 1 Service de Médecine Interne A, Hôpital Charles Nicolle, Tunis, Tunisie; 2 Service ORL, Hôpital Charles Nicolle, Tunis, Tunisie

**Keywords:** maladie de Behçet, endocardite infectieuse, Candida albicans

## Image en medicine

La maladie de Behçet est une vascularite inflammatoire associant plusieurs manifestations cliniques dont la pathogénie reste encore mal élucidée. L'association à une endocardite infectieuse tricuspidienne est rarement rapportée dans la littérature. Patient âgé de 39 ans, était suivi pour une maladie de Behçet depuis 7 ans; ce diagnostic a été posé devant l'association d'une aphtose bipolaire, de lésions de pseudofolliculite et d'un test pathergique positif. Il a été admis pour prise en charge d'une thrombose de la veine fémorale gauche. A l'admission, il avait en plus d'un gros membre inférieur gauche douloureux une aphtose buccale surinfectée associée à une mycose linguale. Il a été traité par amoxicilline-acide clavulinique, métronidazole et Fungysone en suspension orale associés à un traitement anticoagulant. L’évolution était marquée par l'apparition au dixième jour de traitement d'une fièvre à 40°C sans autres signes accompagnateurs. Il n'avait pas de souffle à l'auscultation cardiaque ni de signes évocateur d'une infection cutanée ou urinaire ou digestive. A la biologie, il avait un syndrome inflammatoire et une anémie inflammatoire. L’échographie transthoracique a montré une endocardite tricuspidienne avec présence d'une végétation de 16 mm flottant dans le ventricule droit. Le patient a été mis initialement sous l'association Vancomycine, Imipinème et Amikacine. L’évolution était marquée par la persistance de la fièvre. Devant la positivité de l'antigénémie à Candida albicans et la présence d'anticorps anti- Candida albicans, le diagnostic d'endocardite à Candida albicans a été retenu. Le fluconazole a été mis en route avec une bonne évolution clinique, biologique et échographique.

**Figure 1:**
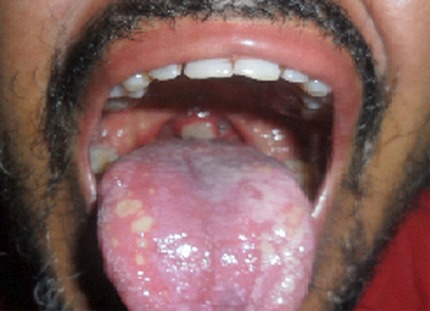
Plusieurs lésions d'aphtose surinfectée de la langue et de la luette de 3 mm à 15 mm de grand axe

